# Brazilian governmental database linkage to correct the municipalities underreported cases in a time-dependent cluster analysis on COVID-19.

**DOI:** 10.23889/ijpds.v7i3.2092

**Published:** 2022-08-25

**Authors:** Anderson Ara, Jonatas Espirito Santo, Jackson Conceição, Marcos Ennes Barreto, Lilia Carolina Carneiro Costa, Rafael Felipe da Silva Souza, Rosemeire Leovigildo Fiaccone, Mauricio Lima Barreto, Maria Yury Travassos Ichihara

**Affiliations:** 1 CIDACS-Fiocruz, Brazil

## Objectives

Covid-19 databases have detailed information about each affected person in Brazil, but it has flaws in counting the number of cases, which are underreported. We aimed to construct and correct the cases dataset by linking different sources of data observations to study the pandemic evolution in Brazilian municipalities.

## Approach

Using the electronic Unified Health System (e-SUS), a public and governmental database, we calculated the pandemic curves of COVID-19 cases. We applied the following approaches to investigate data anomalies a) to perform a descriptive analysis and compare these results with a non-governmental database using Dynamic Time Warping distance; b) to verify and correct municipalities data anomalies linking to other public governmental database namely National Council of Health Secretaries (CONASS) with e-SUS. c) To apply a K-means DTW Barycenter Averaging in clustering analysis to describe the general behaviors of pandemic in Brazilian Municipalities.

## Results

Around 10% records of cases in the e-SUS public governmental database were underreported. After the linkage and the data updating procedure, the time-dependent clustering analysis presents no anomalies and more interpretable results. The clustering analysis provided eight different behaviors of COVID-19 curves of cases. The degree of intensity for prevalence and incidence rates were identified according to eight clusters from the lowest to highest.

## Conclusion

Using the matching procedure based on Dynamic Time Warping distance to correct the municipalities unreported cases, we provided a richer dataset to support a clustering time dependent analysis to characterize the Pandemic evolution in Brazil. These results may be explored in future deprivation social studies.

